# Environmental, developmental, and genetic factors differentially affect rapid ethylene growth response kinetics in dark-grown *Arabidopsis thaliana* hypocotyls

**DOI:** 10.1371/journal.pone.0359974

**Published:** 2026-10-06

**Authors:** Arkadipta Bakshi, Vipsa Patel, Andrew Ward, Mahbuba Ferdous, Brad M. Binder

**Affiliations:** 1 Genome Science and Technology Program, University of Tennessee, Knoxville, Tennessee, United States of America; 2 Department of Biochemistry & Cellular and Molecular Biology, University of Tennessee, Knoxville, Tennessee, United States of America; University of Massachusetts Amherst, UNITED STATES OF AMERICA

## Abstract

Studies on dark-grown wild-type *Arabidopsis thaliana* seedlings have demonstrated that the hypocotyls respond to the application and removal of the hormone ethylene with stereotypical growth kinetics. This includes a rapid decrease in growth rate when ethylene is applied that stabilizes at a low growth rate approximately 75 min after the addition of saturating levels of ethylene. Hypocotyl growth rate recovers to pre-treatment levels approximately 80 minutes after removal of ethylene. The growth of plants is influenced by environmental conditions that can vary between laboratories as well as genetic backgrounds and developmental factors. To address the importance of these variables on ethylene responses, experiments were conducted to determine the effects of composition of growth medium, seed size, seedling age, and accession on ethylene growth response kinetics. These variables led to only minor alterations in growth inhibition kinetics when ethylene was added with the largest effect being an acceleration in growth inhibition when nutrients were excluded from the medium. By contrast, growth recovery kinetics upon ethylene removal showed more plasticity. Sucrose accelerated growth recovery, whereas other environmental variables delayed recovery compared to controls. Other variables that are often overlooked, such as seed size and seedling age, also affected growth recovery where older seedlings recovered more slowly and plants from larger seeds recovered faster than plants from smaller seeds. Accessions from more northern latitudes tended to recover faster than those from more southern latitudes. Our data indicate that these factors need to be carefully controlled for optimal reproducibility of responses to ensure accurate interpretation of data and make sure important traits are not masked.

## Introduction

Ethylene is a hormone that affects the development, growth, and stress responses of plants [1]. One response to ethylene, the triple response, occurs when dark-grown eudicot seedlings are treated with ethylene for several days. In *Arabidopsis thaliana* this response is characterized by reduced elongation of the hypocotyl and root, increased diameter of the hypocotyl, and increased apical hook curvature [2–4]. Changes in physiological responses from specific mutations are often used to determine the role of a gene. For instance, the decreased growth of the roots and hypocotyls that occurs during the triple response are easily quantified and have been used as an assay for several decades to screen for and characterize ethylene-related mutants leading to much of what we know about ethylene biosynthesis and signal transduction [5,6].

More nuanced information has been obtained by examining the rapid growth responses of hypocotyls of dark-grown *A. thaliana* seedlings to the application and removal of ethylene. These studies uncovered new details about ethylene receptor function and sub-functionalization [7–14], down-stream signaling components [15–22], apical hook control [23,24], high sensitivity to ethylene [25], and inhibitors of ethylene perception [12,26]. However, to determine the roles of genes in physiological responses, it is necessary to minimize environmental and developmental variables that affect physiological responses. For instance, various factors such as nutrient availability, seed size, and seedling age affect root growth and gravitropism in *A. thaliana* [27,28]. Without properly controlling for these variables, the roles of a gene can be masked or misinterpreted.

With this in mind, we studied the effects of genetic background, seedling age, seed size, and growth medium on rapid ethylene growth response kinetics to the application and removal of ethylene. Results from this study indicate that growth inhibition kinetics when ethylene is added are less sensitive to changes in these variables than growth recovery kinetics after removal of ethylene. Our data show that these factors need to be carefully controlled to avoid introducing variability in the results that could affect interpretation of the data.

## Results

### Effects of growth medium on ethylene growth response kinetics

To achieve better experimental reproducibility and understanding about the factors that control ethylene responses, we examined the effects of variations in the growth media on ethylene growth response kinetics. We defined control medium as 0.8% (w/v) plant agar (Sigma, A4800), half-strength Murashige and Skoog basal medium with Gamborg’s vitamins at pH 5.7 with no further additions. For controls, we used 250−300 µm diameter Columbia seeds and analyzed them 2 days after light treatment. Under these conditions, the hypocotyl growth rate prior to application of ethylene was 0.27 ± 0.02 mm h^-1^ (Table 1). Ethylene response kinetics to a 2 hour exposure to 10 ppm ethylene were similar to prior studies conducted under the same conditions [17]. This included two phases of growth inhibition when ethylene was added (Fig 1A). The first phase was a rapid deceleration in growth followed by a plateau in growth rate. Following this plateau was a second, slower onset deceleration in growth which reached a new steady-state growth rate approximately 75 min after application of ethylene. Growth recovery was first detectable approximately 35 min after removal of ethylene as determined by cumulative sum control (CUSUM) chart analysis to examine deviations in trends of growth. Full recovery to pre-treatment growth rates occurred approximately 80 min after ethylene removal. The growth rate remained higher than pre-treatment rates for several hours. In contrast to the ethylene-induced changes in growth rate, the growth rate in the absence of added ethylene remained stable with only minor changes for 8 h as previously reported [8].

**Table 1:** Growth rates prior to treatment with ethylene^a^.

Accession	Growth medium	Seed diameter (µm)	Seedling age (days)	Average growth rate ± SEM (mm h^-1^)	Statistics^c^
Columbia	control^b^	250-300	2	0.27 ± 0.02	
Columbia	control + AVG	250-300	2	0.41 ± 0.02	***
Columbia	control + sucrose	250-300	2	0.25 ± 0.03	ns
Columbia	0.8% (w/v) plant agar	250-300	2	0.29 ± 0.01	ns
Columbia	0.8% (w/v) Phytoagar	250-300	2	0.27 ± 0.01	ns
Columbia	control at pH 4.5	250-300	2	0.33 ± 0.02	ns
Columbia	control at pH 7.0	250-300	2	0.30 ± 0.01	ns
Columbia	control at pH 9.5	250-300	2	0.10 ± 0.02	***
Columbia	control	> 300	2	0.27 ± 0.03	ns
Columbia	control	< 250	2	0.12 ± 0.02	***
Columbia	control	250-300	3	0.21 ± 0.02	ns
Columbia	control	250-300	4	0.13 ± 0.01	***
WS-2	control	250-300	2	0.27 ± 0.01	ns
Ler-2	control	250-300	2	0.26 ± 0.02	ns
Sha	control	250-300	2	0.23 ± 0.03	ns
Cvi-0	control	250-300	2	0.25 ± 0.01	ns

^a^Time-lapse imaging was used to determine the growth rates of seedlings as described in the materials and methods. The basal growth rate was determined during the 1 hour prior to ethylene treatment. Control conditions (row 1) involved using 250–300 µm diameter Columbia seeds and analyzing them 2 days after light treatment. The composition of the medium, seed size, seedling age, and accession were varied as shown.

^b^Control medium was half-strength Murashige- Skoog basal medium with Gamborg’s vitamins, 0.8% (w/v) plant agar, pH 5.7.

^c^The data were analyzed by ANOVA for statistical differences from control conditions as designated in row 1. (ns, not significant *P* > 0.05); *** adjusted *P* < 0.001).

**Fig 1:**
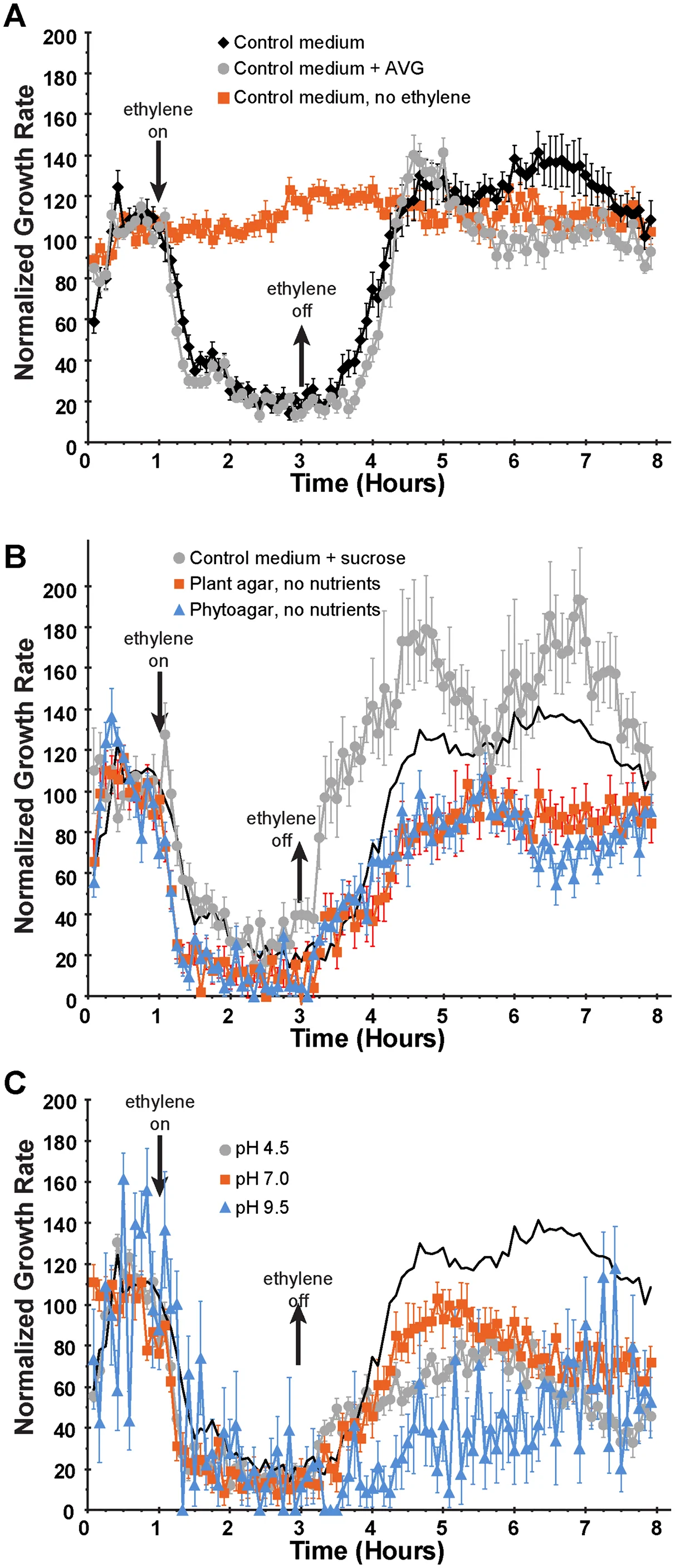
The effects of medium composition on ethylene growth response kinetics. Hypocotyl growth rates were recorded for 1 h in the absence of exogenous ethylene, at which time 10 ppm ethylene was applied for 2 h. Recovery after ethylene removal was measured for 5 h. Data from each seedling were normalized to the growth rate in air prior to ethylene treatment and represents the average ± SEM of at least 6 seedlings. Half-strength Murashige and Skoog basal medium with Gamborg’s vitamins, 0.8% (w/v) plant agar, pH 5.7 is the control medium for this study. **A)** Ethylene growth response kinetics in the absence (control) and presence of 5 µM AVG compared to the growth rates of seedlings without the addition of ethylene (no AVG). **B)** Ethylene growth response kinetics in the presence of sucrose and absence of added nutrients on either plant agar or Phytoagar. **C)** Effects of pH on ethylene growth response kinetics. B, C) Control data from Fig 1A are shown as a solid black line for comparison.

Many studies examining ethylene responses include L-α-(2-amino ethoxyvinyl)-glycine (AVG) to block endogenous ethylene production. Inclusion of 5 µM AVG significantly increased hypocotyl growth rate in the absence of ethylene (Table 1). This likely reflects inhibition of endogenous ethylene production. Despite the higher basal growth rate, the inhibition kinetics were slightly accelerated in the presence of AVG (Fig 1A). The latency for growth recovery was slightly delayed from controls where it took 50 min for growth recovery to be detectable by CUSUM. By contrast, the rate of recovery was approximately 50% faster than controls. These differences during growth recovery led to a similar time of growth recovery to pre-treatment growth rates compared to controls. In the presence of 5 µM AVG, there was a growth rate overshoot that then returned to pre-treatment levels approximately 2.75 h after removal of ethylene. In the absence of AVG, the growth rate remained elevated for a longer time.

Sucrose is a common additive to plant medium to provide energy and promote growth. Addition of 0.8% (w/v) sucrose did not significantly alter the hypocotyl growth rate under these conditions (Table 1) and growth inhibition kinetics in the presence of sucrose were similar to controls (Fig 1B). By contrast, after removal of ethylene, the seedlings grown on sucrose recovered much faster than controls taking approximately 25 min to reach pre-treatment growth rates. These seedlings then had an overshoot in the growth rate followed by an oscillation in growth rate. Growth recovery after removal of ethylene was first detectable approximately 20 min sooner than controls.

Because of these results with sucrose and prior observations showing that availability of nutrients affects gravitropism of *A. thaliana* [27,28], we were curious to know if low nutrient availability would affect ethylene growth response kinetics. When seedlings were grown on 0.8% (w/v) plant agar with no other additions, the growth rate before application of ethylene was not significantly affected (Table 1). However, the seedlings showed faster growth inhibition and an apparent single phase of growth inhibition with no plateau in growth rate prior to full growth inhibition (Fig 1B). A similar acceleration in inhibition kinetics was previously described for seedlings lacking the *EIN3-Binding F-Box1* gene, which is important in ethylene signaling [16]. After removal of ethylene, recovery took longer than control conditions. Different types of agar vary in purity and the components present. We therefore also examined responses using 0.8% (w/v) Phytoagar (A20300, Research Products International Corp.) and obtained similar results to those with plant agar.

We also examined the effects of pH on ethylene growth inhibition and recovery kinetics by comparing responses at pH 4.5, 5.7, 7.0, and 9.5 (Fig 1C). Hypocotyl growth rates in the absence of ethylene were similar for all pHs except at pH 9.5, where the growth rate was significantly reduced (Table 1). Responses to the application of ethylene at pH 4.5 and 7.0 were similar to responses at pH 5.7. Recovery after removal of ethylene took longer at both pH 4.5 and 7.0 with no overshoot in growth rate and at later timepoints, incomplete recovery to pre-treatment growth rates. At pH 9.5, it was difficult to accurately assess growth inhibition kinetics because of noise in the data. With this limitation, it appears that the kinetics of inhibition are similar to what is seen at other pH values. Growth recovery after ethylene removal was strongly impacted at pH 9.5 and did not recover to the pre-treatment rate in the time-course of the experiments. The slow recovery was due to both a longer latency (3 h longer than controls) and a slower rate of recovery (approximately 9-fold slower than controls).

### Effects of seed size on ethylene growth response kinetics

*A. thaliana* seed size affects various traits such as germination time, root growth, root gravitropism, and flowering time [27,28]. We therefore wished to know whether ethylene response kinetics were affected by seed size. To examine this, we mechanically sieved seeds from a single batch of seeds to give large seeds (> 300 µm in diameter), medium seeds (250–300 µm in diameter) as used for all other experiments described in this study, and small seeds (< 250 µm in diameter). The hypocotyl growth rates prior to treatment with ethylene were similar for seedlings from large and medium seeds but were statistically reduced for small seeds (Table 1). The growth inhibition kinetics were similar for seedlings from all three sized seeds (Fig 2A). However, the recovery kinetics after removal of ethylene were different between the three populations. The seedlings from large seeds recovered the fastest and had a large overshoot in growth rate compared to medium seeds. The large seed seedling growth rates plateaued at a higher value than the pre-treatment rate. The plants from small seeds took longer to recovery than the other two seed populations. This slower recovery was largely due to a longer delay for growth recovery to start where recovery started at approximately 95 min after removal of ethylene as determined by CUSUM.

**Fig 2:**
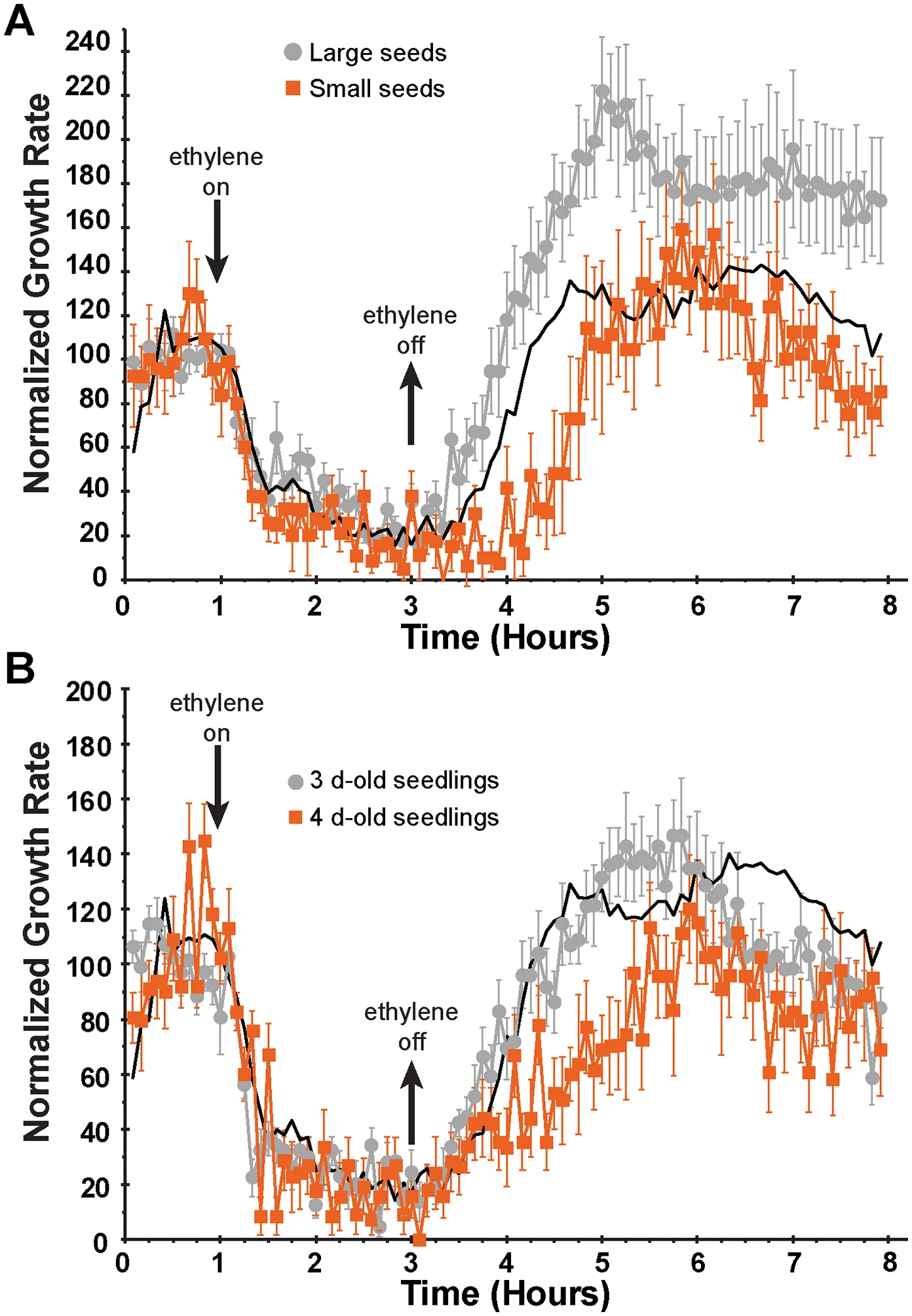
The effects of seed size and seedling age on ethylene growth response kinetics. Hypocotyl growth rates were recorded for 1 h in the absence of exogenous ethylene, at which time 10 ppm ethylene was applied for 2 h. Growth recovery after ethylene removal was measured for 5 h. Data from each seedling were normalized to the growth rate in air prior to ethylene treatment and represents the average ± SEM of at least 7 seedlings. **A)** Comparison of ethylene growth response kinetics of seedlings from large (> 300 µm in diameter) and small seeds (< 250 µm in diameter). **B**) Ethylene growth response kinetics of 3-day-old and 4-day-old seedlings. Control data of 2-day-old seedlings from medium-sized seeds (250-300 µm in diameter) from Fig 1A are shown as a solid black line for comparison.

### Effects of seedling age on ethylene growth response kinetics

Our prior studies examined ethylene growth kinetics at 2 d after light treatment [7,25] and this was the control timing used for other experiments in the current study. Under the conditions used in this study in darkness, seedlings germinate at approximately 32 h after light treatment [29]. We wished to determine if seedling age affected ethylene response and recovery kinetics. We therefore examined the ethylene growth response kinetics of seedlings 3 d and 4 d after light treatment (Fig 2B). The hypocotyl growth rate of 4 d seedlings in the absence of ethylene was lower for these older seedlings and likely reflects lower seed storage molecules for growth. The growth inhibition kinetics were similar for the three populations. The 3 d seedlings had recovery kinetics similar to 2 d seedlings. By contrast, the 4 d seedlings took longer to recover. Initially, the 4 d seedlings had similar recovery kinetics to the controls. But, at approximately 1 h after ethylene removal, the rate of recovery slowed to approximately 33% of the controls.

### Ethylene growth response kinetics of different accessions

The above results indicate that environmental and developmental factors can affect ethylene growth response kinetics. Since ethylene growth response kinetics vary between species [17], we also wished to determine if different *A. thaliana* accessions have different responses to the application and removal of ethylene. We chose accessions from widely disparate geographical regions and used seeds 250−300 µm in diameter. All of the accessions had similar growth rates in air (Table 1) and similar growth inhibition kinetics when ethylene was applied (Fig 3). However, there were differences in growth recovery when ethylene was removed. Wassilewskija (WS-2) and Landsberg erecta (Ler-2) recovered to pre-treatment growth rates slightly faster than Columbia and had an overshoot in growth rate. By contrast, Cape Verde Islands (Cvi-0) and Shahdara (Sha) recovered slower and plateaued at growth rates slightly lower than the pre-treatment rates. When examining these recovery times relative to latitude of origin, accessions from more southern latitudes tended to take longer to recover than those from more northern latitudes (Fig 4A). No such correlation occurred relative to the longitudes of origin (Fig 4B). The rate of recovery was slower (Fig 4C) and the latency time for recovery to start was longer (Fig 4D) for more southern accessions.

**Fig 3:**
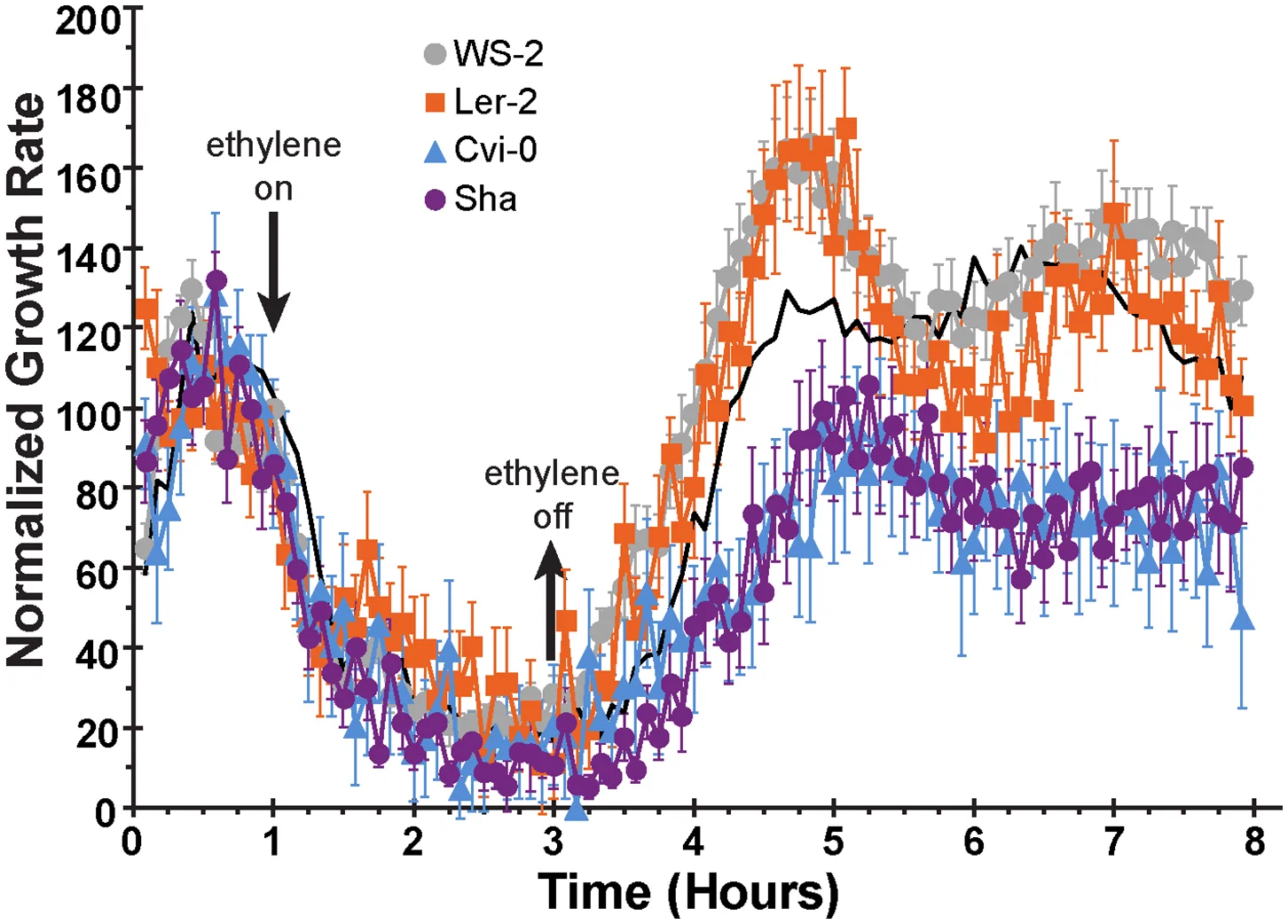
Ethylene growth response kinetics of different *A. thaliana* accessions. Hypocotyl growth rates of different accessions were recorded for 1 h in the absence of exogenous ethylene, at which time 10 ppm ethylene was applied for 2 h. Recovery after ethylene removal was measured for 5 h. Data from each seedling were normalized to the growth rate in air prior to ethylene treatment and represents the average ± SEM of at least 13 seedlings. Control data from Fig 1A for Columbia seedlings are shown as a solid black line for comparison.

**Fig 4:**
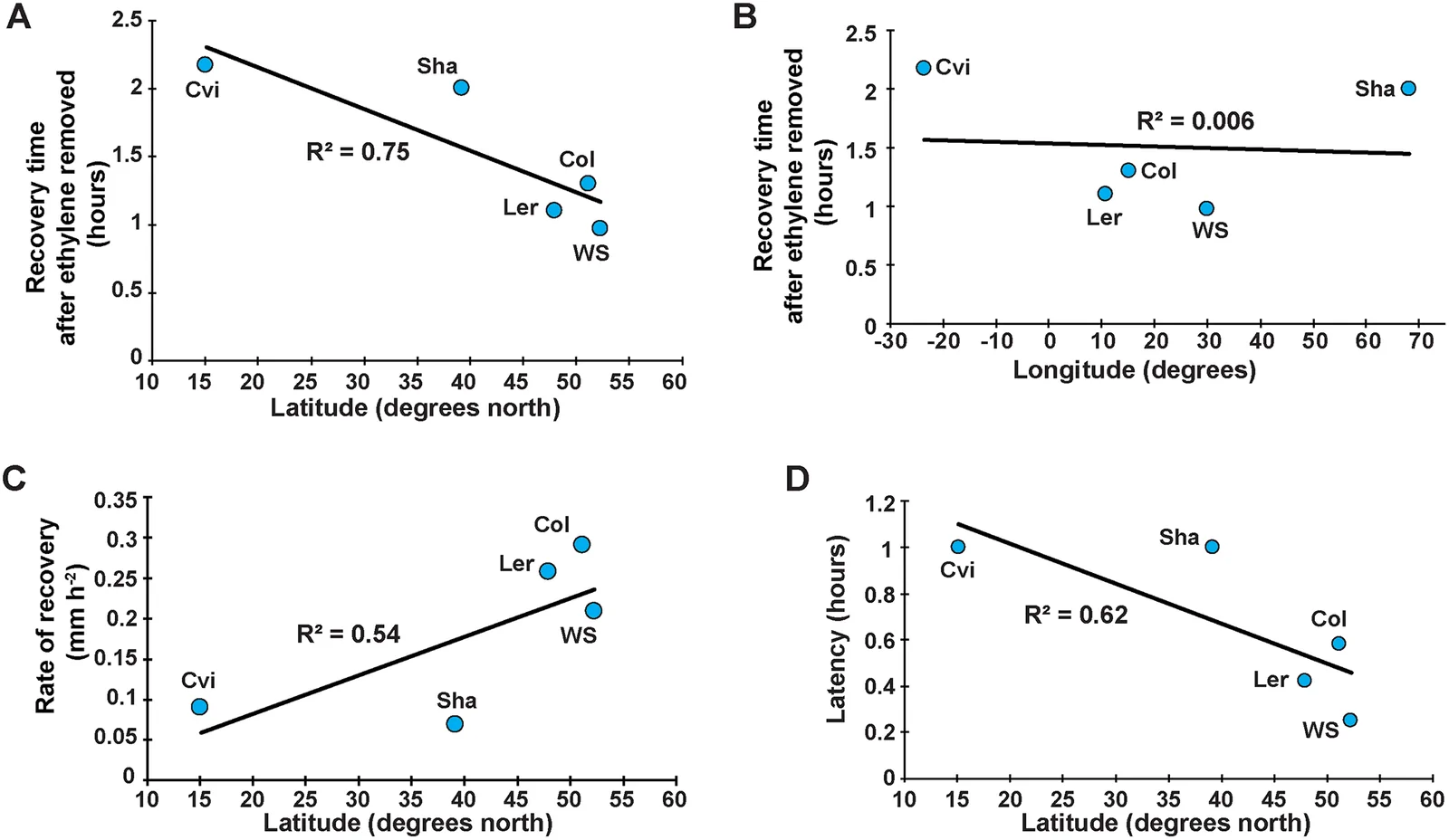
Growth recovery after ethylene removal correlates with latitude of origin. The approximate latitude and longitude of where the accessions were collected was determined. The time for growth recovery to reach pre-treatment growth rate for each accession is plotted against the **A)** latitudes and **B)** longitudes of origin. **C)** The rate of growth recovery after removal of ethylene calculated during the linear phase of recovery and **D)** the latency time before growth recovery started as determined by CUSUM analyses is plotted against the latitudes of origin. The linear phases of recovery were determined by inspection.

## Discussion

In this study, we demonstrate that environmental, developmental, and genetic factors affect hypocotyl growth and ethylene growth response kinetics of dark-grown *A. thaliana* hypocotyls. These variables generally cause only minor alterations in growth inhibition kinetics when ethylene is added. By contrast, growth recovery kinetics show more variability. This is consistent with prior studies showing that growth recovery kinetics after ethylene removal are more highly impacted by genetic factors than inhibition kinetics [7,11,13,15–17,19–22,30]. The basis for this higher plasticity in response is not known.

With a few exceptions, hypocotyl growth rates are consistent across the variations in environmental conditions used in this study, yet growth recovery after removal is often affected. This indicates that there is not a correlation between basal hypocotyl growth rate prior to application of ethylene and recovery of growth after removal of ethylene. For instance, application of AVG leads to approximately a 50% increase in hypocotyl growth rate which, no doubt, is due to the inhibition of ethylene biosynthesis by this chemical. This is consistent with increased growth in response to application of AVG that was previously measured with end-point analysis [31]. However, AVG causes only subtle changes in the time for growth recovery which might be due to altered ethylene biosynthesis, but could also be due to other effects of AVG such as reduced auxin biosynthesis [32]. Exogenous sucrose did not affect the growth rate in the absence of ethylene, but accelerated growth recovery. In contrast, growth recovery of seedlings in the absence of nutrients took longer than controls, even though the basal growth rate in the absence of ethylene was unchanged. A similar lack of correlation between basal growth rate and recovery kinetics after removal of ethylene was previously observed, where loss of specific ethylene receptors or several other components of ethylene signaling, slowed down growth recovery, but had little effect on growth rates in the absence of ethylene [7,16].

Nutrient content of the medium also affects growth recovery. In the absence of nutrients, growth recovery is delayed, even though basal growth before application of ethylene is minimally affected. In contrast, sucrose leads to much faster growth recovery after removal of ethylene. Higher carbon availability for energy is one explanation for faster recovery in the presence of sucrose. However, a confounding factor is that exogenous sucrose leads to increased ethylene biosynthesis by plants [33,34], which is predicted to lead to slower growth and growth recovery. Another complicating factor is that there is a complex interplay between sucrose and ethylene signaling to regulate growth and development of *A. thaliana* [34–38]. This makes it difficult to model the mechanisms by which exogenous sucrose affects growth recovery.

Varying pH between 4.5 and 7.0 does not significantly alter hypocotyl growth rate. It is only at the extremely high pH of 9.5 that the growth rate is significantly reduced. By contrast, variations in these external variables slows down hypocotyl growth recovery after ethylene is removed. Delays in growth recovery are seen when seedlings are grown at pH values that diverge from pH 5.7. Although the most extreme delay is seen at pH 9.5, delays in growth recovery also occur at pH 7.0 and 4.5 compared to pH 5.7. The mechanisms underlying these effects of pH on growth recovery are unknown. Since cell wall loosening is important for growth and is affected by pH [39], these effects of pH potentially involve alterations in apoplastic pH to impact cell wall loosening. The observation that growth rates in air are unaffected by most changes in pH argues against this idea. Ethylene growth response kinetics are affected by the levels of another plant hormone, gibberellin [17] and extracellular pH affects gibberellin accumulation [40,41] pointing to a potential role for gibberellin in mediating the differences observed in growth recovery at different pHs.

Hypocotyl growth rates are slower in seedlings maintained in darkness for 4 d compared to the 2 d usually used. Smaller seeds also give rise to seedlings with lower growth rates. It is likely that these lower growth rates are due to reduced levels of seed storage oils and proteins for energy since experiments were conducted in darkness with no external source of carbon. Growth recovery after ethylene removal takes longer with older seedlings and seedlings from small seeds. Whether this slower recovery is due to lower seed resources is unclear since the characteristics of the slower growth recovery are different for small seed seedlings compared to older seedlings. For plants from small seeds, the longer time for growth recovery is due to a longer latency time before recovery starts, whereas, for older seedlings it is due to a slower rate of growth recovery. Thus, developmental factors affect ethylene growth recovery kinetics, but may do so via different mechanisms.

Natural variation in *A. thaliana* has led to differences in many traits such as leaf and root architecture, flowering time, disease and stress resistance, and seed dormancy and has been used to explore differences in physiological responses in plants [42–44]. In this study, we show that ethylene growth inhibition kinetics upon application of ethylene are fairly similar between the different accessions examined. By contrast, the time for growth recovery after removal of ethylene differs between these accessions. Given that there is variation in ethylene growth response kinetics between species [17], this natural variation between accessions of *A. thaliana* is not surprising. Accessions from southern latitudes took longer to recover than those from farther north. It is unknown if this is a general feature or just applies to the accessions studied. It is also unclear what survival advantages might be conferred by faster growth recovery versus slower recovery. Differences in growth recovery might reflect variations in the optimal growth conditions for each accession. For instance, nutrient availability in the growth medium can alter phenotypic plasticity [28] and we see clear effects of nutrients in the medium on growth recovery rates of Columbia. This raises the possibility that the different accessions have differing nutrient requirements that affect growth recovery. If true, it is possible that the slower recovery of Cvi-0 and Sha can be accelerated to resemble the other accessions under different nutrient conditions. Another thing to consider is that the time for growth recovery is impacted by alterations in the ethylene receptors and other components of ethylene signal transduction [7,11,13,15,16,19–22]. Therefore, the differences in growth recovery between these accessions may be due to variations in the level of one or more component of ethylene signaling.

This study demonstrates that various factors can measurably alter rapid ethylene growth response kinetics, especially growth recovery kinetics after removal of ethylene. This sensitivity of growth recovery kinetics to various environmental, developmental, and genetic factors adds a level of complexity that needs to be addressed when linking specific genes to alterations in ethylene growth kinetics. For example, a mutation that delays growth recovery could be doing so as a direct result of the mutation. However, if the mutation alters seed size or nutrient requirements, the alterations in ethylene responses might be indirect.

The choice of specific conditions for a given study ultimately needs to be determined by the goals of the study. Some factors such as seed accession, pH, and medium composition are obvious factors to control. Other variables, such as seed size and seedling age, are not generally considered, but based on our results should be controlled for. It is also important to note that the composition of the medium has a large effect on ethylene response kinetics. This should be taken into consideration when designing experiments since some effects of ethylene can be partially masked or antagonized by external sugars including sucrose and glucose [45–47], indicating that important alterations in responses could be missed under specific conditions. Overall, our data show the need for carefully controlling various variables to most accurately assess the roles of genes in ethylene growth responses.

## Materials and methods

All experiments were carried out on *A. thaliana*. Cvi-0 (CS6675), Sha (CS929), and Ler-2 (CS8581) seeds were obtained from the Arabidopsis Biological Resource Center (ABRC). Accessions were chosen to represent a wide range of regions for the native population (Col and Ler, Germany; Cvi, Cave Verde Islands; Sha, Tajikistan; WS, Russia). In general, formerly described methods were used for time-lapse imaging and data analysis [7].

### Seedling preparation and growth conditions

Briefly, *A. thaliana* seeds were surface sterilized for 30 s with 70% (v/v) ethanol, dried on sterile filter paper, and then placed on agar plates. For controls, 250–300 µm diameter Columbia seeds were plated on 0.8% (w/v) plant agar plates (A4800, Sigma) containing half-strength Murashige and Skoog basal medium with Gamborg’s vitamins, pH 5.7. Seeds were cold-treated for at least 2 d to break dormancy and light treated under white light for at least 2 h to synchronize germination. Unless otherwise noted, time-lapse imaging was carried out 2 d after light treatment. Variations in composition of the medium, seed size, seedling age, and accession are noted in the results section.

### Measurement of hypocotyl growth rates

For all experiments, images of seedlings growing in treatment chambers were acquired every 5 min for 8 h in darkness using infrared illumination. Each treatment chamber consisted of a vertically orientated round agar plate sealed with a lid that allowed for continuous gas flow as previously described [48]. Gas flow was kept at 100 mL min^-1^ throughout the experiment resulting in a chamber response time for steady-state equilibration of less than 40 seconds. After a 1 h treatment with ethylene-free air to establish basal growth rates, 10 ppm ethylene was introduced for 2 h, followed by 5 h of ethylene-free air to study growth recovery. Image analysis and growth rate calculations were carried out as previously described [7]. Briefly, we calculated the growth rate for each 5-minute increment using Microsoft Excel and these data were normalized to the average growth rate in ethylene-free air as determined from measurements made prior to application of ethylene. In some experiments, the growth rates in the absence of added ethylene were determined for 8 h and normalized to the growth rates in the first hour of the measurements. Data were plotted from at least 4 separate experiments from 4 different time-lapse imaging setups and represent the average ± SEM at each time point. Each line drawn in the time-course plots is the two point moving average determined by Microsoft Excel.

### Statistical analyses

Statistical differences in the average growth rate in air were determined with ANOVA using GraphPad Prism ver. 9.3.1. The latency for growth recovery after removal of ethylene was determined using cumulative sum control (CUSUM) chart analysis to evaluate the growth rate trends and calculate the time where the trends diverged between the control and experimental seedlings [49]. CUSUM analyses were conducted using Excel.

## Supporting information

S1 Data Underlying numerical data. (XLSX)

## References

[1] Abeles FB, Morgan PW, Saltveit ME. Ethylene in Plant Biology. Academic Press: San Diego. 1992.

[2] Guzmán P, Ecker JR. Exploiting the triple response of Arabidopsis to identify ethylene-related mutants. Plant Cell. 1990;2(6):513–23. doi: 10.1105/tpc.2.6.513 2152173 PMC159907

[3] Bleecker AB, Estelle MA, Somerville C, Kende H. Insensitivity to Ethylene Conferred by a Dominant Mutation in Arabidopsis thaliana. Science. 1988;241(4869):1086–9. doi: 10.1126/science.241.4869.1086 17747490

[4] Kieber JJ, Rothenberg M, Roman G, Feldmann KA, Ecker JR. CTR1, a negative regulator of the ethylene response pathway in Arabidopsis, encodes a member of the raf family of protein kinases. Cell. 1993;72(3):427–41. doi: 10.1016/0092-8674(93)90119-b 8431946

[5] Binder BM. Ethylene signaling in plants. J Biol Chem. 2020;295(22):7710–25. doi: 10.1074/jbc.REV120.010854 32332098 PMC7261785

[6] Azhar BJ, Zulfiqar A, Shakeel SN, Schaller GE. Amplification and Adaptation in the Ethylene Signaling Pathway. Small Methods. 2019;4(8). doi: 10.1002/smtd.201900452

[7] Binder BM, O’malley RC, Wang W, Moore JM, Parks BM, Spalding EP, et al. Arabidopsis seedling growth response and recovery to ethylene. A kinetic analysis. Plant Physiol. 2004;136(2):2913–20. doi: 10.1104/pp.104.050369 15466220 PMC523353

[8] Binder BM, O’Malley RC, Wang W, Zutz TC, Bleecker AB. Ethylene stimulates nutations that are dependent on the ETR1 receptor. Plant Physiol. 2006;142(4):1690–700. doi: 10.1104/pp.106.087858 17071649 PMC1676061

[9] Binder BM, Zutz TC, O’Malley RC, Wang W, Bleecker AB. Ethylene stimulates nutations of etiolated Arabidopsis hypocotyls that are dependent on the ETR1 receptor. Advances in Plant Ethylene Research. Springer Netherlands. 2007. p. 3–8. doi: 10.1007/978-1-4020-6014-4_1

[10] Gao Z, Wen C-K, Binder BM, Chen Y-F, Chang J, Chiang Y-H, et al. Heteromeric interactions among ethylene receptors mediate signaling in Arabidopsis. J Biol Chem. 2008;283(35):23801–10. doi: 10.1074/jbc.M800641200 18577522 PMC2527101

[11] Kim H, Helmbrecht EE, Stalans MB, Schmitt C, Patel N, Wen C-K, et al. Ethylene receptor ETHYLENE RECEPTOR1 domain requirements for ethylene responses in Arabidopsis seedlings. Plant Physiol. 2011;156(1):417–29. doi: 10.1104/pp.110.170621 21386032 PMC3091048

[12] McDaniel BK, Binder BM. ethylene receptor 1 (etr1) Is Sufficient and Has the Predominant Role in Mediating Inhibition of Ethylene Responses by Silver in Arabidopsis thaliana. J Biol Chem. 2012;287(31):26094–103. doi: 10.1074/jbc.M112.383034 22692214 PMC3406693

[13] Bakshi A, Wilson RL, Lacey RF, Kim H, Wuppalapati SK, Binder BM. Identification of Regions in the Receiver Domain of the ETHYLENE RESPONSE1 Ethylene Receptor of Arabidopsis Important for Functional Divergence. Plant Physiol. 2015;169(1):219–32. doi: 10.1104/pp.15.00626 26160962 PMC4577405

[14] Aman S, Swain S, Dutta E, Abbas S, Li N, Shakeel SN, et al. Modulation of plant growth and development through altered ethylene binding affinity of the ethylene receptor ETR1. BMC Plant Biol. 2025;25(1):436. doi: 10.1186/s12870-025-06469-y 40186127 PMC11971883

[15] Potuschak T, Vansiri A, Binder BM, Lechner E, Vierstra RD, Genschik P. The exoribonuclease XRN4 is a component of the ethylene response pathway in Arabidopsis. Plant Cell. 2006;18(11):3047–57. doi: 10.1105/tpc.106.046508 17085683 PMC1693942

[16] Binder BM, Walker JM, Gagne JM, Emborg TJ, Hemman G, Bleecker AB. The Arabidopsis EIN3-Binding F-Box Proteins, EBF1 and 2 Have Distinct but Overlapping Roles in Regulating Ethylene Signaling. The Plant Cell. 2007;19:509–23. 10.1105/tpc.106.048140 PMC1867343 17307926

[17] Kim J, Wilson RL, Case JB, Binder BM. A comparative study of ethylene growth response kinetics in eudicots and monocots reveals a role for gibberellin in growth inhibition and recovery. Plant Physiol. 2012;160(3):1567–80. doi: 10.1104/pp.112.205799 22977279 PMC3490611

[18] Merchante C, Brumos J, Yun J, Hu Q, Spencer KR, Enríquez P, et al. Gene-specific translation regulation mediated by the hormone-signaling molecule EIN2. Cell. 2015;163(3):684–97. doi: 10.1016/j.cell.2015.09.036 26496608

[19] Rai MI, Wang X, Thibault DM, Kim HJ, Bombyk MM, Binder BM, et al. The ARGOS gene family functions in a negative feedback loop to desensitize plants to ethylene. BMC Plant Biol. 2015;15:157. doi: 10.1186/s12870-015-0554-x 26105742 PMC4478640

[20] Binder BM, Kim HJ, Mathews DE, Hutchison CE, Kieber JJ, Schaller GE. A role for two-component signaling elements in the Arabidopsis growth recovery response to ethylene. Plant Direct. 2018;2(5):e00058. doi: 10.1002/pld3.58 31245724 PMC6508545

[21] Azhar BJ, Abbas S, Aman S, Yamburenko MV, Chen W, Müller L, et al. Basis for high-affinity ethylene binding by the ethylene receptor ETR1 of Arabidopsis. Proc Natl Acad Sci U S A. 2023;120(23):e2215195120. doi: 10.1073/pnas.2215195120 37253004 PMC10266040

[22] Park HL, Seo DH, Lee HY, Bakshi A, Park C, Chien Y-C, et al. Ethylene-triggered subcellular trafficking of CTR1 enhances the response to ethylene gas. Nat Commun. 2023;14(1):365. doi: 10.1038/s41467-023-35975-6 36690618 PMC9870993

[23] Zádníková P, Petrásek J, Marhavy P, Raz V, Vandenbussche F, Ding Z, et al. Role of PIN-mediated auxin efflux in apical hook development of Arabidopsis thaliana. Development. 2010;137(4):607–17. doi: 10.1242/dev.041277 20110326

[24] Vandenbussche F, Petrásek J, Zádníková P, Hoyerová K, Pesek B, Raz V, et al. The auxin influx carriers AUX1 and LAX3 are involved in auxin-ethylene interactions during apical hook development in Arabidopsis thaliana seedlings. Development. 2010;137(4):597–606. doi: 10.1242/dev.040790 20110325

[25] Binder BM, Mortimore LA, Stepanova AN, Ecker JR, Bleecker AB. Short-term growth responses to ethylene in Arabidopsis seedlings are EIN3/EIL1 independent. Plant Physiol. 2004;136(2):2921–7. doi: 10.1104/pp.104.050393 15466219 PMC523354

[26] Pirrung MC, Bleecker AB, Inoue Y, Rodriguez FI, Sugawara N, Wada T. Ethylene receptor antagonists: strained alkenes are necessary but not sufficient. Chem Biol. 2008;15:313–21. 10.1016/j.chembiol.2008.02.018 18420138

[27] Elwell AL, Gronwall DS, Miller ND, Spalding EP, Brooks TLD. Separating parental environment from seed size effects on next generation growth and development in Arabidopsis. Plant Cell Environ. 2011;34(2):291–301. doi: 10.1111/j.1365-3040.2010.02243.x 20955226

[28] Brooks TLD, Miller ND, Spalding EP. Plasticity of Arabidopsis root gravitropism throughout a multidimensional condition space quantified by automated image analysis. Plant Physiol. 2010;152(1):206–16. doi: 10.1104/pp.109.145292 19923240 PMC2799357

[29] Wilson RL, Kim H, Bakshi A, Binder BM. The Ethylene Receptors ETHYLENE RESPONSE1 and ETHYLENE RESPONSE2 Have Contrasting Roles in Seed Germination of Arabidopsis during Salt Stress. Plant Physiol. 2014;165(3):1353–66. doi: 10.1104/pp.114.241695 24820022 PMC4081342

[30] Christians MJ, Gingerich DJ, Hansen M, Binder BM, Kieber JJ, Vierstra RD. The BTB ubiquitin ligases ETO1, EOL1 and EOL2 act collectively to regulate ethylene biosynthesis in Arabidopsis by controlling type-2 ACC synthase levels. Plant J. 2009;57(2):332–45. doi: 10.1111/j.1365-313X.2008.03693.x 18808454 PMC2807402

[31] Larsen PB, Chang C. The Arabidopsis *eer1* mutant has enhanced ethylene responses in the hypocotyl and stem. Plant Physiol. 2001;125(2):1061–73. doi: 10.1104/pp.125.2.1061 11161061 PMC64905

[32] Soeno K, Goda H, Ishii T, Ogura T, Tachikawa T, Sasaki E, et al. Auxin biosynthesis inhibitors, identified by a genomics-based approach, provide insights into auxin biosynthesis. Plant Cell Physiol. 2010;51(4):524–36. doi: 10.1093/pcp/pcq032 20234049

[33] Ellison CT, Vandenbussche F, Van Der Straeten D, Harmer SL. XAP5 CIRCADIAN TIMEKEEPER regulates ethylene responses in aerial tissues of Arabidopsis. Plant Physiol. 2011;155(2):988–99. doi: 10.1104/pp.110.164277 21163961 PMC3032482

[34] Mu X-R, Tong C, Fang X-T, Bao Q-X, Xue L-N, Meng W-Y, et al. Feedback loop promotes sucrose accumulation in cotyledons to facilitate sugar-ethylene signaling-mediated, etiolated-seedling greening. Cell Rep. 2022;38(11):110529. doi: 10.1016/j.celrep.2022.110529 35294871

[35] Haydon MJ, Mielczarek O, Frank A, Román Á, Webb AAR. Sucrose and Ethylene Signaling Interact to Modulate the Circadian Clock. Plant Physiol. 2017;175(2):947–58. doi: 10.1104/pp.17.00592 28778922 PMC5619894

[36] Kwon Y, Oh JE, Noh H, Hong S-W, Bhoo SH, Lee H. The ethylene signaling pathway has a negative impact on sucrose-induced anthocyanin accumulation in Arabidopsis. J Plant Res. 2011;124(1):193–200. doi: 10.1007/s10265-010-0354-1 20514508

[37] Smalle J, Haegman M, Kurepa J, Van Montagu M, Straeten DV. Ethylene can stimulate Arabidopsis hypocotyl elongation in the light. Proc Natl Acad Sci U S A. 1997;94(6):2756–61. doi: 10.1073/pnas.94.6.2756 11038610 PMC20163

[38] Gibson SI, Laby RJ, Kim D. The sugar-insensitive1 (sis1) mutant of Arabidopsis is allelic to ctr1. Biochem Biophys Res Commun. 2001;280(1):196–203. doi: 10.1006/bbrc.2000.4062 11162499

[39] Cosgrove DJ. Plant Cell Wall Loosening by Expansins. Annu Rev Cell Dev Biol. 2024;40(1):329–52. doi: 10.1146/annurev-cellbio-111822-115334 38724021

[40] Kramer EM. How far can a molecule of weak acid travel in the apoplast or xylem? Plant Physiol. 2006;141(4):1233–6. doi: 10.1104/pp.106.083790 16896235 PMC1533949

[41] Rizza A, Tang B, Stanley CE, Grossmann G, Owen MR, Band LR, et al. Differential biosynthesis and cellular permeability explain longitudinal gibberellin gradients in growing roots. Proc Natl Acad Sci U S A. 2021;118(8):e1921960118. doi: 10.1073/pnas.1921960118 33602804 PMC7923382

[42] Koornneef M, Alonso-Blanco C, Vreugdenhil D. Naturally occurring genetic variation in Arabidopsis thaliana. Annu Rev Plant Biol. 2004;55:141–72. doi: 10.1146/annurev.arplant.55.031903.141605 15377217

[43] Leventhal L, Ruffley M, Exposito-Alonso M. Planting Genomes in the Wild: Arabidopsis from Genetics History to the Ecology and Evolutionary Genomics Era. Annu Rev Plant Biol. 2025;76(1):605–35. doi: 10.1146/annurev-arplant-071123-095146 39971350

[44] Weigel D. Natural variation in Arabidopsis: from molecular genetics to ecological genomics. Plant Physiol. 2012;158(1):2–22. doi: 10.1104/pp.111.189845 22147517 PMC3252104

[45] Brenya E, Dutta E, Herron B, Walden LH, Roberts DM, Binder BM. Ethylene-mediated metabolic priming increases photosynthesis and metabolism to enhance plant growth and stress tolerance. PNAS Nexus. 2023;2(7):pgad216. doi: 10.1093/pnasnexus/pgad216 37469928 PMC10353721

[46] Zhou L, Jang JC, Jones TL, Sheen J. Glucose and ethylene signal transduction crosstalk revealed by an Arabidopsis glucose-insensitive mutant. Proc Natl Acad Sci U S A. 1998;95(17):10294–9. doi: 10.1073/pnas.95.17.10294 9707641 PMC21502

[47] Yanagisawa S, Yoo S-D, Sheen J. Differential regulation of EIN3 stability by glucose and ethylene signalling in plants. Nature. 2003;425(6957):521–5. doi: 10.1038/nature01984 14523448

[48] Binder BM. Time-Lapse Imaging to Examine the Growth Kinetics of Arabidopsis Seedlings in Response to Ethylene. Methods Mol Biol. 2017;1573:211–22. doi: 10.1007/978-1-4939-6854-1_14 28293848

[49] Duncan AJ. Cumulative Sum Control Charts. 4th ed. New York: Wiley. 1974.

